# Microfluidic vascular models of tumor cell extravasation

**DOI:** 10.3389/fonc.2022.1052192

**Published:** 2022-11-11

**Authors:** Seunggyu Kim, Zhengpeng Wan, Jessie S. Jeon, Roger D. Kamm

**Affiliations:** ^1^ Mechanobiology Lab, Department of Mechanical Engineering, Massachusetts Institute of Technology, Cambridge, MA, United States; ^2^ Biomicrofluidics Lab, Department of Mechanical Engineering, Korea Advanced Institute of Science and Technology, Daejeon, South Korea

**Keywords:** cancer extravasation, microfluidic chip, preclinical disease model, *in vitro* vascular model, pre-metastatic microenvironment

## Abstract

Emerging microfluidic disease models have amply demonstrated their value in many fields of cancer research. These *in vitro* technologies recapitulate key aspects of metastatic cancer, including the process of tumor cell arrest and extravasation at the site of the metastatic tumor. To date, extensive efforts have been made to capture key features of the microvasculature to reconstitute the pre-metastatic niche and investigate dynamic extravasation behaviors using microfluidic systems. In this mini-review, we highlight recent microfluidic vascular models of tumor cell extravasation and explore how this approach contributes to development of *in vitro* disease models to enhance understanding of metastasis *in vivo*.

## Introduction

**Figure 1:**
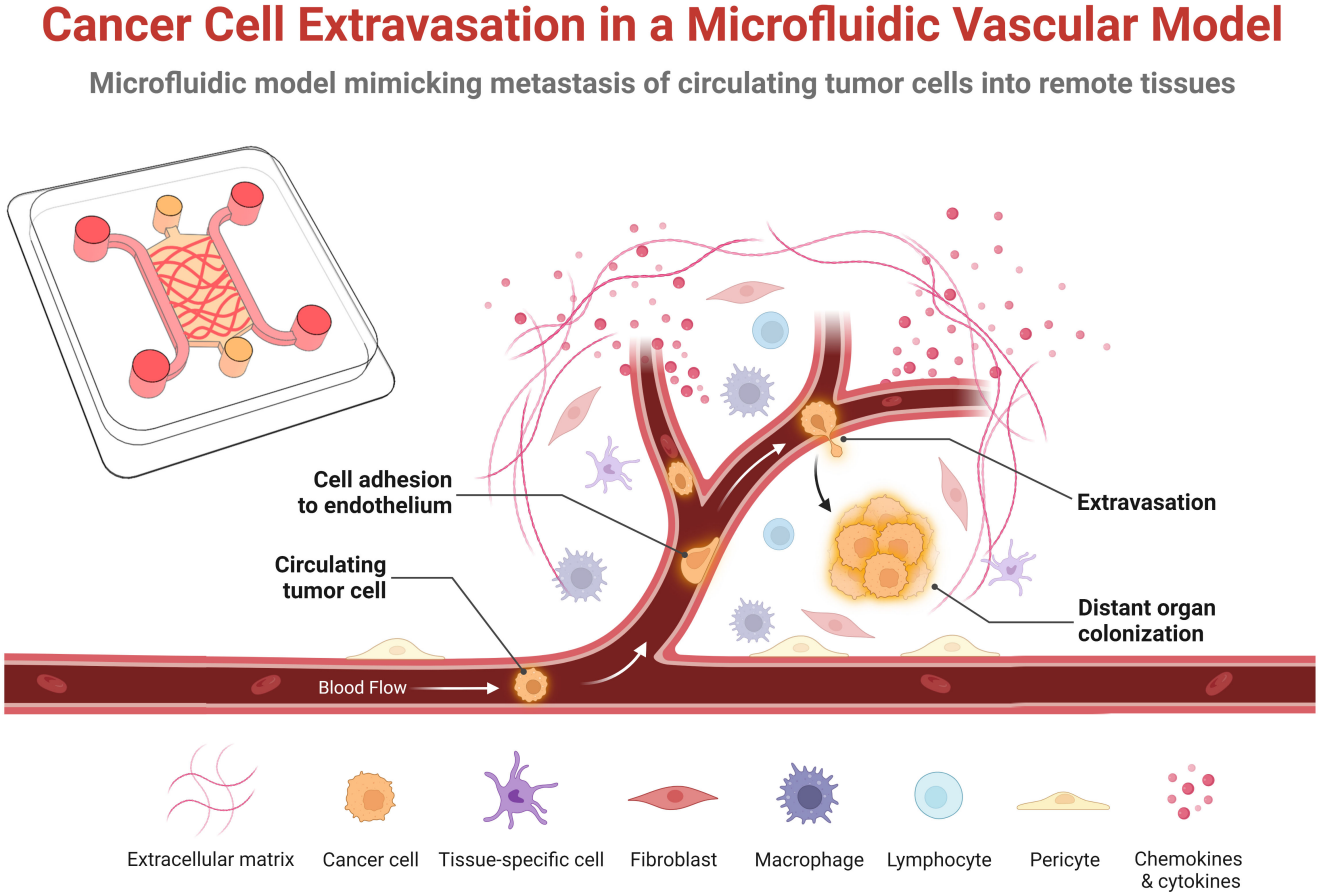
Schematic representation of the key steps of cancer cell extravasation within a microfluidic vascular model. Circulating tumor cells originate from the primary tumor, enter the bloodstream that transports them to a distance organ or tissue where they adhere or become physically trapped in the microvasculature. Some fraction of these survive in the vessel and undergo trans-endothelial migration by extending protrusions across the endothelial monolayer, adhering to the basal membrane and extracellular matrix, and pulling or pushing itself across the endothelial monolayer into the underlying tissue. In the extravascular space, the extravasated tumor cells then either proliferate, die, or enters into a dormant state. This schematic was created with BioRender.com.

Despite advances in cancer treatment such as surgical resection and adjuvant therapy, cancer metastasis still accounts for more than 90% of cancer patients’ deaths (1). Cancer metastasis often begins when tumor cells undergo an epithelial-to-mesenchymal transition (EMT), in which they acquire higher motility and invasiveness and break away from the primary tumor (2). Then, these tumor cells invade surrounding tissues, intravasate to the nearby microvasculature as either single cells or cell clusters, and become circulating tumor cells (CTCs), often aggregating with platelets or monocytes, enabling them to withstand fluid shear stress or anoikis (3) and avoid attack by circulating NK cells. Surviving CTCs can then adhere to the endothelium and extravasate into the distant secondary organ and subsequently progress into metastatic tumors (**Figure 1**). In the secondary organ, factors including the pre-metastatic microenvironment (e.g., resident stromal cells) determines the extravasation sites and plays an important role in metastasis progression (4). Therefore, it is important to explore how the interactions between CTCs and the microenvironment in the secondary organ affect cancer cell extravasation.

Preclinical animal models such as patient-derived xenograft (PDX) mouse models have been established to study cancer stemness and stroma-tumor interactions, predict potential biomarkers, and identify anti-cancer therapeutic strategies (5). PDX models preserve blood vessels and stroma conserved in the surgical tissue, which reflects the unique features of primary tumor and enables a more accurate evaluation of anti-metastatic treatments. Although PDX models have led to enormous advances in anti-cancer therapeutic research, certain inherent challenges are still difficult to address. A major hurdle is the use of immunodeficient mice to overcome transplant rejection, which hampers the studies of the complex roles of the immune system involved in metastasis (6). The applicable types and grades of cancers are limited for successful establishment of PDX models, and the human stroma of xenografts may be replaced by murine components due to a series of tumor passaging, resulting in loss of the characteristics of the original cancer tissue. In addition, access to high-resolution imaging technologies to visualize tumor cell activity in PDX models is another challenge (7), despite the vast capabilities of intravital imaging (8). Finally, a lack of control over various important steps of the metastatic cascade, the presence of non-human cells, and limitations to the use of genetic modifications all point to the need of alternative models.

Microfluidic-based cancer models, also called tumor-on-a-chip models, have emerged as an intermediate and complementary technology positioned between animal models and *in vitro* two-dimensional (2D) models (9–11). A microfluidic three-dimensional (3D) system is defined as a miniaturized cell culture platform engineered to model the functional units of human tissue/organs (12, 13). This advanced technology recapitulates many features of the complex human tumor/host microenvironments by controlling biophysical and biochemical factors in on-demand manners, which enables the reconstitution of key biological processes and disease states (14–16). Numerous reviews have summarized the advances in tumor-on-a-chip models (17–23). Here, we focus on processes occurring in the metastatic site utilizing microfluidic vascular models (**Table 1**).

**Table 1:** Microfluidic vascular models for studying cancer cell extravasation.

Vascular model type(s)	Cocultured parenchymal cell type(s)	Cancer cell line(s)	Highlighted feature	Reference
iPSC-EC MVNs in 3D fibrin hydrogel	Brain pericytes, brain astrocytes	Breast cancer (MDA-MB-231) and its brain metastatic variant (MDA-BrM2a) and lung metastatic variant (MDA-LM2-4175); Breast cancer (SKBR3); Lung carcinoma (A549)	CCL2-CCR2 axis in brain metastasis	(24)
HUVEC monolayer on porous membrane	LFs, macrophages, bronchial epithelial cells	Lung carcinoma (A549)	Extravasated cancer cells inducing damages of tissue-specific cells	(25)
HPMEC monolayer on porous membrane; HBMVEC monolayer on hydrogel	LFs, mononuclear cells, bronchial epithelial cells, brain astrocytes	Lung cancer (PC9) its brain metastatic subpopulations (PC9-BrM1, PC9-BrM2, PC9-BrM3)	Higher extravasation into brain tissue by lung cancer cells expressing AKR1B10	(26)
HUVEC MVNs in 3D fibrin hydrogel	LFs	Breast cancer (MDA-MB-231); Fibrosarcoma (HT1080)	Integrin signaling (Cdk5/Tln1/FAK^S732^) in extravasation	(27)
HUVEC MVNs in 3D fibrin hydrogel	LFs	Mammary epithelial cell (HMLER) and its mesenchymal derivative (NAMEC8R)	EMT promoting extravasation through a podocalyxin-ezrin axis	(28)
HUVEC MVNs in 3D fibrin hydrogel	LFs	Breast cancer (MDA-MB-231, MCF7); Renal carcinoma (SN12C, SN12PM6)	Glycocalyx-mediated adhesion to endothelium and trans-endothelial migration	(29)
HUVEC MVNs in 3D fibrin hydrogel	LFs	Breast cancer (MDA-MB-231, MCF-7); Mammary epithelial cell (MCF-10A)	Hypoxia increasing HIF-1α expression and extravasation rates of cancer cells.	(30)
HUVEC MVNs in 3D fibrin hydrogel	LFs	Breast cancer (MDA-MB-231)	Fluid flow (luminal, trans-endothelial, and/or interstitial)-mediated extravasation	(31)
HUVEC monolayer on 3D collagen hydrogel		Breast cancer (MDA-MB-231, MCF-7)	Substrate stiffness affecting MMP-9 expression and extravasation of invasive cancer cells	(32)
HUVEC MVNs in 3D fibrin hydrogel	LFs	Breast cancer (MDA-MB-231); Melanoma (A375, A375-MA2)	Inflamed neutrophils promoting extravasation *via* localization of IL-8	(33)
HDMVEC monolayer on 3D collagen hydrogel		Breast cancer (MDA-MB-231)	Formation of pre-metastatic niche by monocytes and macrophages	(34)
HUVEC MVNs in 3D fibrin hydrogel	LFs	Breast cancer (MDA-MB-231); Melanoma (MDA-MB-435)	Monocytes reducing extravasation rates in a paracrine manner	(35)
HUVEC MVNs in 3D fibrin hydrogel	LFs	Breast cancer (MDA-MB-231)	Increased extravasation in the presence of neutrophils and platelets	(36)
HUVEC MVNs in 3D collagen/fibrin hydrogel	LFs	Breast cancer (MDA-MB-231)	Neutrophils promoting protrusions and trans-endothelial migration of cancer cells by activating endothelium	(37)
HUVEC in 3D bone decellularized matrix	BM-MSCs	Breast cancer (MDA-MB-231)	Perfusion culture reducing colonization and drug sensitivity of adhered cancer cells	(38)
HDMVEC monolayer on 3D collagen hydrogel	Osteoblasts, MSCs, LFs	Breast cancer (MDA-MB-231) and its metastatic subpopulations (bone tropic and lung tropic MDA-MB-231)	Organ-specific parenchymal cells dictating selective extravasation of subpopulations of breast cancer cells	(39)
HUVEC MVNs in 3D fibrin hydrogel	Osteo-differentiated BM-MSCs, myoblasts	Bone seeking clone of the breast cancer cell (BOKL clone MDA-MB-231); Mammary epithelial cell (MCF-10A)	Organ-specific breast cancer extravasation into bone- or muscle-mimicking microenvironments	(40)
iPSC-EC monolayer on 3D collagen/fibrin hydrogel		Breast cancer (MDA-MB-231, MCF-7, Hs-578T)	Cancer-vascular crosstalk promoting cancer extravasation	(41)
HUVEC MVNs in 3D fibrin hydrogel; HUVEC monolayer on 3D collagen/fibrin hydrogel	LFs	Breast cancer (MDA-MB-231); Melanoma (A375-MA2); Mouse mammary carcinoma (4T1)	Integrin β1-mediated trans-endothelial migration of cancer cells	(42)
HUVEC monolayer on 3D fibrin hydrogel	LFs	Prostate cancer (PC3)	Recapitulating several key metastatic steps of tumor cells	(43)
Rat BMVEC monolayer on 3D collagen hydrogel	Astrocytes	Breast cancer (MDA-MB-231); Lung carcinoma (A549); Melanoma (M624); Hepatocellular carcinoma (BEL-7402)	Mimicking extravasation of human breast, lung, and melanoma cells across the BBB	(44)
HUVEC monolayer on 3D Matrigel		Breast cancer (MDA-MB-231)	Real-time monitoring of single cancer cell extravasation under SDF-1α gradient	(45)
HUVEC MVNs in 3D collagen/fibrin hydrogel		Breast cancer (MDA-MB-231)	Weakened endothelial glycocalyx at the side of cancer cell extravasation visualized with a lectin stain	(46)
HUVEC monolayer on 3D collagen hydrogel		Breast cancer (MDA-MB-231, MCF-7)	Impairment of cancer cell extravasation using an EGFR targeting drug	(47)
HUVEC monolayer on 3D collagen hydrogel		Breast cancer (MDA-MB-231, LM2-4175)	Real-time monitoring of cell trajectorial dynamics during the trans-endothelial migration	(48)
HUVEC monolayer on 3D collagen/Matrigel hydrogel	Osteocyte-like cells	Breast cancer (MDA-MB-231)	Reduced extravasation rates and distance of cancer cells under flow-applied bone-mimicking microenvironment	(49)
HUVEC monolayer on 3D collagen hydrogel		Breast cancer (MDA-MB-231, MCF-7, Hs-578T)	Hyaluronan-rich pericellular matrix promoting cancer cell extravasation	(50)
HUVEC monolayer on Matrigel-coated membrane of micropillars		Breast cancer (MDA-MB-231)	Real-time monitoring of adhesion and trans-endothelial migration of cancer cells under continuous physiological flow	(51)
HUVEC MVNs in 3D fibrin hydrogel	LFs	Colon cancer (SW620)	Culture tumor cells under hydronic pressure-driven flow, SW620 formed cell aggregates before extravasation.	(52)
HUVEC monolayer on 3d Matrigel		Breast cancer (MDA-MB-231)	Attracted by CXCL12, cancer cells need 10 hours to extravasate through endothelium.	(53)
HCMEC/D3 monolayer on porous membrane	Astrocytes	Breast cancer (MDA-231-BR, MDA-MB-231, JIMT-1)	Astrocytes secrete Dkk-1 promoting tumor cell extravasation.	(54)
HLSEC monolayer on porous membrane	LF, hepatocytes, liver stellate cells	Breast cancer (MDA-MB-231, MCF-7); Glioblastoma (U-87MG); Pancreatic cancer (PANC-1, Capan-1); Mammary epithelial cell (MCF-10A)	Breast cancer-derived extracellular vesicles activating endothelium and promoting cancer cell extravasation.	(55)
iPSC-HBMEC monolayer on collagen-condensed porous membrane	Astrocytes	Breast cancer (MDA-MB-231, MCF-7)	Breast cancer cell extravasation across the BBB on a collagen-layered porous membrane	(56)

HUVEC, human umbilical vein endothelial cell; iPSC-EC, induced pluripotent stem cell-derived endothelial cell; HDMVEC, human dermal microvascular endothelial cell; EMT, epithelial-mesenchymal transition; MVN, microvascular network; HIF-1α, hypoxia-inducible factor 1 α; IL-8, interleukin 8; HPMEC, human pulmonary microvascular endothelial cell; HBMVEC, human brain microvascular endothelial cell; LF, lung fibroblast; BM-MSC, bone marrow-derived mesenchymal s7tem cell; BBB, blood-brain barrier; SDF-1α, stromal derived factor-1α; HCME3/D3, human cerebral microvascular endothelial cell; HLESC, human liver sinusoidal endothelial cell.

## Microfluidic vascular models to study tumor cell extravasation

One approach used in modeling the process of extravasation *in vitro* utilizes microfluidic vascular models (57). There are three representative types of on-chip endothelial models, namely, a self-assembled microvascular network (MVN), an endothelial-lined channel, and a planar endothelial barrier (58). In summary, the self-assembled MVN (59) or endothelial-lined channel (60) can be created *via* the proliferation and maturation of vascular cells that are embedded within the 3D extracellular matrix (ECM) or seeded on the gel-liquid interface, respectively. In these designs, on-chip implant of microliter-scale hydrogel is realized with microstructures such as hydrophilic surface rail, sacrificial gel, micro-posts, or channel height differences (61–64). The planar endothelial barrier is typically formed by vascular cell proliferation onto a microporous polymer membrane flanked by microchannels. (65). Utilizing the membrane requires the examination of whether several parameters including pore size, pore density, and thickness hinder the transport of molecules and cells while maintaining tissue barrier function with a sufficient contact area. To overcome these challenges, other semi-permeable materials such as dried ECM film or electrospun nanofibers have been integrated into the microfluidic system (66, 67). Notably, the engineered vascular models have different characteristics as follows. For instance, the 3D MVN has the smallest vessel diameters (~ 15-50 μm) compared to those of others, recapitulating *in vivo* microvasculature (68, 69). Given the importance of ECM in tumor extravasation and colonization, the presence of ECM that encapsulates MVNs can provide the experimental opportunity for studying the protease activity of tumor cells during ECM invasion and adaption of tumor cells at the secondary microenvironment. The planar endothelial barrier model allows the acquisition of conditioned medium in extravascular region and the measurement of transepithelial electrical resistance (TEER) across the endothelium, which are limited in the MVN model.

Cancer cells are seeded in these lumenized channels or vasculature where they interact with the host microenvironment, become physically lodged in the small vessels or adhere to the endothelium, and extravasate across the endothelial monolayer (70). Because of its high versatility and robust reproducibility, microfluidic vascular models have been used for pre-clinical assays, such as studies of anti-metastatic drug responses and immunotherapy. Previous reviews have summarized tumor extravasation studies using microfluidic vascular models (57, 71–75), thus we only highlight here the recently published works.

## Adhesion molecules in tumor cell extravasation

Cell-cell and cell matrix adhesion plays a central role in the arrest and transmigration of tumor cells. Earlier studies in 3D, self-assembled MVNs have investigated the role of various integrins during extravasation, identifying the importance of the α3β1 and α6β1 subunits, implicating laminin in the endothelial basement membrane as a key factor in extravasation (42). Other adhesion molecules have been identified in the initial adhesion of a tumor cell to the endothelium. For example, in a series of studies, the role of the glycocalyx, a common surface coating of glycoproteins and proteoglycans, has been studied (76). The endothelial glycocalyx, including hyaluronic acid (HA), heparan sulfate, and chondroitin sulfate, has traditionally been considered as a repulsive barrier that prevents cell adhesion (77). Recently, an *in vitro* microfluidic vascular model identified a mechanism in which tumor cells prepared an adhesive vascular niche for subsequent tumor cell adhesion by depositing tumor HA on the apical surface of the endothelium (29). Notably, tumor cells arrested on such an adhesive vascular niche through their CD44v binding to the deposited HA. Genetic and pharmacological inhibition of CD44 abrogated such adhesion. These results suggested that the cancer glycocalyx may be a promising target for therapeutic intervention for metastasis.

Beside glycocalyx, focal adhesion signaling pathway is also important for tumor cell arrest and extravasation (78). Gilardi et al. investigated the cyclin-dependent kinase 5 (Cdk5)/Talin 1 (Tln1)/focal adhesion kinase (FAK) axis in breast cancer and fibrosarcoma extravasation using a three-gel channel microfluidic device, where the microvasculature was in the center and stromal cells are seeded in the left and right gel channels (27). Knocking down Tln1, FAK, or Cdk5 in tumor cells reduced vascular adhesion and trans-endothelium migration. Particularly, inhibitor experiments showed that FAK S732 phosphorylation was required for tumor cell extravasation, which may be explained by phosphorylated-S732 dependent pERK expression in nuclei.

## Physical factors in tumor cell extravasation

Throughout the process of metastasis, tumor cells are subjected to a variety of physical stressors such as fluid shear stress (FSS) (79), and their progress is influenced by mechanical factors such as ECM fiber orientation and substrate stiffness (80, 81). To investigate the effects of fluid flows on cancer extravasation, breast cancer cells have been perfused into an *in vitro* microfluidic MVN model, controlling the intravascular and transmural (trans-endothelial) pressure differences to produce physiological luminal, transmural, and interstitial flows (31). These breast cancer cells extravasated in a single cell manner. When applied with luminal flow (up to ~500 μm/s), cancer cell extravasation efficiencies were significantly increased compared to static control, independent of transmural flow rate. Notably, transmural flow increased both the speed of tumor cell trans-endothelial migration and migration in the surrounding matrix. The directionality of tumor cells under flows was also assessed: intravascular adherent tumor cells traveled predominantly in the direction of luminal flow, whereas extravasated tumor cells showed random migration patterns. This work suggests the importance of applying physiological luminal flow when studying extravasation *in vitro*.

Another microfluidic platform constructed by a sequential edge-guided patterning method was developed to study tumor cell intravasation and extravasation (52). In this study, colon cancer cells, SW620, were perfused into MVNs and cultured under a flow that was driven by a hydrostatic pressure differential between medium channels. Unlike the breast cancer cells mentioned above (31), SW620 formed micro-tumors attached to the endothelium and extravasated from the vessels. Future work on the mechanism is still under investigation. Different from the self-organized MVNs mentioned above, the Goetz group used a human umbilical vein EC (HUVEC)-coated channel to mimic vasculature and investigated endothelial remodeling by FSS. They found that flow upregulated VEGFR signaling in ECs and that blocking VEGFR signaling reduced EC remodeling and tumor cell extravasation (82).

Besides FSS, substrate stiffness, as another biophysical cue, was recently investigated for its role in regulating cancer cell extravasation (32). Breast cancer cell lines, MCF7 and MDA-MB-231, were precultured on fibronectin-coated polydimethylsiloxane (PDMS) substrates of different stiffness and then added to an EC monolayer in a microfluidic device to evaluate their extravasation rate. Tumor cells precultured on the stiff substrate had the highest extravasation rate probably due to the elevated MMP9 expression in tumor cells. The same group, later utilized the same EC monolayer system to evaluate the extravasation rate of breast cancer cells treated with an EGFR-targeting anti-cancer drug, Cetuximab. The reduced extravasation rate with Cetuximab treatment was associated with altered cell physical features with decreased actin, vinculin, and myosin II expression (47).

## Tissue-specific models for tumor cell extravasation study

Cancers typically exhibit organ-specificity in their metastatic cascades, known as “organotropism”, in which cancers tend to predominantly spread to a distant organ or show sequential organ-specific colonization (83). Organ-specific metastasis is likely regulated by multiple factors, including circulation patterns, tumor-intrinsic factors, metabolic changes, immune landscapes of target organs, and the interaction between tumor cells and the secondary organ microenvironment (84). Although the underlying mechanisms of non-random distributions of metastatic cancers have been not fully understood, clinical evidence has indicated clear patterns of organ-specific metastasis (85). Given the complexity involved in organ-specific metastasis, 3D microfluidic cell culture systems have advantages in recapitulating various features of the host microenvironment by employing organ- or tissue-specific cell types in a miniaturized 3D assay compared to conventional systems. As a representative study, a 3D *in vitro* blood-brain barrier (BBB) model was used to investigate the mechanisms brain-specificity in multiple cancer types (24). This study confirmed previous studies in mice that identified ST6GALNAC5 as an important factor in brain selectivity (86). It also showed that, in addition, astrocytes in brain parenchyma directly promoted cancer cell transmigration through C-C motif chemokine ligand 2 (CCL2) secretion (24). The BBB model was composed of induced pluripotent stem cell (iPSC)-derived ECs (iPSC-ECs), brain pericytes, and astrocytes, reconstituting key morphological and functional characteristics of the brain capillaries (i.e. cellular organization, expression of junctional and transporter proteins, vascular permeability, and deposition of the basement membrane and glycocalyx). Another group recently developed a blood-brain niche system using a two-channel microfluidic device separated by a porous membrane (54). The bottom of the device was filled with human astrocytes in collagen gel to recapitulate a brain microenvironment. ECs were seeded as a monolayer on the membrane to serve as a barrier. MDA-231-BR tumor cells were perfused into the vessel and extravasated by the attraction of astrocytes themselves or astrocytes-conditioned medium. The secretome analysis of ECs and astrocytes identified that Dickkopf-related protein 1 (Dkk-1) was elevated in astrocytes when stimulated with MDA-231-BR. Treating tumor cells with Dkk-1 upregulated FGF-13, PLCB1, and MYC gene expression, which are key components in Ras, PI3K-MAPK, and Wnt signaling pathways. Neutralizing Dkk-1 and knocking down FGF-13 in tumor cells were able to reduce the extravasation rate. This work identified DKK-1 function in the brain metastatic niche using a vascularized microfluidic device. Recently, an omentum-on-a-chip model was developed by including ECs, adipocytes, mesothelial cells, and tumor cells to mimic tumor cell invasion from the peritoneal cavity to the sub-peritoneal space (87). Although this model was not used to study metastasis from the vasculature, it could also be used for tissue specific tumor cell extravasation studies since it contained a perfusable MVN.

## Immune cell function in tumor cell extravasation

During metastasis, tumor cells encounter numerous types of immune cells, i.e. platelets, monocytes, neutrophils, NK cells, and lymphocytes (88). The immune cells are considered cooperative or antagonistic for the cancer progression depending on the nature of their complex interactions with microenvironmental factors and tumor cells (89). Previous studies have demonstrated the function of neutrophils (33), monocytes (35), and macrophages (34) in tumor cell extravasation using the microfluidic vascular models. Recently, another report explored the contribution of neutrophils and platelets in tumor cell extravasation (36). Neutrophils and platelets were co-cultured on the MDA-MB-231 monolayer for 24 hours before being perfused into the MVNs, of which the platelets promoted tumor cell mesenchymal phenotypes. Co-perfusing neutrophils and platelets with tumor cells increased the tumor cell extravasation rate. Treating the system with a clinically-approved integrin β3-targeting antiplatelet drug impaired platelet aggregation and activation, decreased mesenchymal marker expression in tumor cells, downregulated phosphorylation of VE-cadherin in ECs, lowered the permeability of the MVNs, all contributing to a reduction in tumor cell extravasation rates. This work demonstrated the capability of utilizing a microfluidic vascular platform to study immune cell and clinical drug functions in metastasis.

## Extravasation of tumor cell clusters and other recent studies

It has been found that clusters of circulating tumor cells (CTCs) have a greater propensity to cause metastases than single cells (3). Several studies have therefore included perfused tumor cell clusters, in addition to single CTCs, into microfluidic vasculature to mimic circulating tumor cluster transportation *in vivo* (59, 90). Recently, a two-step seeding method was developed to generate perfusable MVNs with physiological-like small diameters, where tumor cell clusters (containing 2-4 cells) were trapped and tracked to monitor extravasation (91). Another vessel-on-a-chip platform was developed to study CTC and endothelium interactions (43). Prostate cancer (PC3) single cells or clusters were perfused through an EC-coated channel with a diameter over 200 µm and extravasated under HGF stimulation. Tumor cells extravasated without visibly disrupting the integrity of the endothelium, but tumor clusters destroyed the integrity of the vessels in a brutal and faster extravasation manner.

3D MVNs were established in microfluidics to compare the extravasation rate of lung cancer cells with different EMT phenotypes (92). A549 holoclone cells with an epithelial and stem-like phenotype, and A549 paraclone cells with a mesenchymal phenotype, were perfused into MVNs and cultured for 24 hours. Paraclones extravasated across the vessels whereby holoclones remained inside the microvasculature. Another vessel-on-a-chip model was developed to demonstrate breast cancer cell MDA-MB-231 extravasation in the presence of a chemoattractant, CXCL12 (53). The mold of this device was laser-cut and the gel channel and medium channel were separated with a porous membrane. Comparing the migration rate of cancer cells with or without an EC monolayer towards CXCL12 over 24 hours, the authors discovered that MDA-MB-231 cells required at least 10 hours to cross the endothelium. The underlying mechanisms were left for future studies.

An iPSC-derived vessel-on-a-chip model was employed to explore cancer-vascular interactions during cancer cell extravasation (41). The implanted iPSC-ECs formed a single lumen structure allowing intravascular perfusion of tumor cells. Within hours of co-culture, the perfused tumor cells adhered to the endothelium and extravasated out of the vessel. MDA-MB-231 breast cancer cells upregulated endothelial ICAM-1 expression through paracrine signaling. In contrast, extravasating cancer cells reduced endothelial collagen IV deposition *via* cell-cell physical interactions. In addition, inflammatory cytokines such as MMP-3, IL-6, and IL-8 were significantly upregulated in cancer-vascular co-culture conditions, resulting in increased vascular permeability and correspondingly increased trans-endothelial cancer cell migration, which were consequently inhibited by the addition of therapeutic inhibitors of these cytokines.

## Challenges, future perspectives, and conclusions

Since the concept of the organ-on-a-chip has been introduced, numerous studies have demonstrated that microfluidic vascular models could closely mimic key steps of metastasis. Despite many promising contributions of these models, there are still several challenges to overcome.

First, there remain many challenges in tissue engineering of cancer. Although several efforts have been made to robustly generate perfusable vasculature *in vitro* (91, 93, 94), micro-vessels with small diameters, high perfusability, specific tissue-type, and high physiological/pathological relevance are needed. This could be address through the use of primary cells from patients to generate an improved pathological model for personalized medicine. Most studies generated vasculature using model ECs and stromal cells (e.g., HUVECs and normal lung FBs), usually lacking the pathology-associated features of patient cells. Whether and to what extent cancer in one location affects the vasculature in a remote metastatic site, however, remains an open question. In addition, most studies use cancer cell lines. Although these have been shown in many studies to behave in a similar manner to patient cancer cells, some cell lines lose their original cancer characteristics along multiple passaging. Thus, primary tumor cells, especially CTCs/clusters, along with patient-derived vascular cells are recommended to establish better models to mimic metastasis. The use of patient-iPSC-derived cells is a viable alternative for personalized models providing accurate prediction and treatment of diseases. Another challenge of current microfluidic (and other *in vitro*) systems is the choice of culture medium. Most studies used media that favor endothelium survival, but this may compromise physiological/pathological relevance. More efforts are needed to create a new medium that better mimics whole blood.

Second, challenges also arise in the basic engineering of the models. External equipment often connects to microfluidics to apply mechanical stimulations, such as FSS. Such equipment could be a barrier for researchers lacking engineering experience. Continued efforts are needed to develop actuators and new microfluidic systems with high accessibility and compatibility. While miniaturized microfluidic systems have the advantage of requiring only a small number of cells, conventional biological assays often demand a large number of cells. Thus, highly-sensitive assays need to be incorporated into current platforms. Multi-organ microfluidic systems combining two or more organotypic modules are being developed. Currently, modules are connected through a simple microchannel/tubing, which impedes the ability to independently regulate individual modules’ functions. A precisely controlled system should be developed to mimic each organotypic microenvironment while connected through tubing, or better yet, living vasculature.

Regarding experimental techniques, some new directions could be explored in the future. The studies of tumor cell extravasation heavily rely on imaging-based assays, requiring cell labeling, immunostaining, etc. Integrating real-time detection systems into the microfluidic assays could enable continuous tracking of cell dynamics and microenvironmental changes during metastasis. Other imaging-related probes could be integrated to study these dynamic changes, such as chemical or genetic biosensors (calcium sensor, pH sensor, cell membrane tension sensor, kinase phosphorylation FRET sensor) in real time (95). Other imaging techniques could also be utilized, such as super-resolution imaging, traction force microscopy, optical tweezers, and atomic force microscopy (AFM), although some challenges have to be solved first (95). For example, a new open-top design will be needed to allow the AFM probe to access vessels or tumor cells. Besides imaging, other assays could also be used, such as Luminex ELISA for secretome analysis, Seahorse XF for real-time metabolic analysis, and next-generation sequencing for gene expression analysis.

One barrier to using RNA sequencing is how to collect extravasated cells and separate them from the non-extravasated ones. Microneedle systems might be able to extract single extravasated tumor cells from matrix, but the throughput would be low. Another strategy is to ablate endothelium on demand using chemical inducible cell death tools (96). For example, ECs could be engineered to express the inducible cell death system and further form a perfusable vasculature. After tumor cells are perfused into the vasculature and extravasation has occurred, then the non-extravasated tumor cells could be eliminated from vasculature by a high intravascular flow. Next, the chemical agent to specifically induce EC death could be introduced, dead cell debris removed by washing, and the extravasated tumor cells remaining in the stroma collected by matrix digestion and sorted. These tumor cells could be used for both bulk and single-cell RNA-seq to study the chances during extravasation.

As for new tissue type platforms, liver is among the most highly metastasized tissues, but there are only a few liver tissue-specific microfluidic vascular models. One recent study generated liver-specific endothelium using liver sinusoidal ECs in a membrane based microfluidic device, and demonstrated that transforming growth factor β1 (TGFβ1) in the breast cancer-derived extracellular vesicles (EVs) increased cancer cell adhesion to liver endothelium (55). However, more work is still needed to study tumor cell extravasation in the liver microenvironment. Similarly, although some *in vitro* studies focus on tumor cell interaction with lymphatic vessels, none address the potential for tumor cell extravasation from lymphatic vessels.

Finally, there needs to be a balance between new technology development and studies of the underlying biology of tumor cell extravasation. More collaborations are needed between the developers of the technology and the oncologists and cancer biologists probing the mechanisms of metastasis, for example, how tumor-resident intracellular bacteria, which promote CTC survival (97), would regulate tumor cell extravasation.

In summary, microfluidic vascular models have emerged that recapitulate many aspects of *in vivo* cancer metastasis. These engineering efforts have and continue to show enormous value in elucidating the basic mechanisms of metastasis and are pointing the way toward personalized medicine. As these approaches gain wider acceptance, they will also help to reduce, and perhaps someday replace, animal experiments. The future is bright, but there remains much to do.

## Author contributions

All authors conceptualized, wrote and reviewed the manuscript under supervision by RK and JJ. All authors contributed to the article and approved the submitted version.

## Funding

This work was supported by the US National Cancer Institute (U01 CA214381 and U54 CA261694) and by a grant of the Korea Health Technology R&D Project through the Korea Health Industry Development Institute (KHIDI), funded by the Ministry of Health and Welfare, Republic of Korea (no. HI20C0589).

## Conflict of interest

RK is the co-founder of and holds a significant financial interest in AIM Biotech, a company that produces microfluidic devices. RK also receives research support from Amgen, Novartis, Boehringer Ingelheim, GSK, AbbVie and Roche.

The remaining authors declare that the research was conducted in the absence of any commercial or financial relationships that could be construed as a potential conflict of interest.

## Publisher’s note

All claims expressed in this article are solely those of the authors and do not necessarily represent those of their affiliated organizations, or those of the publisher, the editors and the reviewers. Any product that may be evaluated in this article, or claim that may be made by its manufacturer, is not guaranteed or endorsed by the publisher.
